# Are self-ligating brackets related to less formation of *Streptococcus mutans* colonies? A systematic review

**DOI:** 10.1590/2176-9451.19.1.060-068.oar

**Published:** 2014

**Authors:** Leonard Euler Andrade Gomes do Nascimento, Margareth Maria Gomes de Souza, Angela Rita Pontes Azevedo, Lucianne Cople Maia

**Affiliations:** 1 Visiting professor, Federal University of Piauí (UFPI).; 2 Full professor, Department of Pediatric Dentistry and Orthodontics, Federal University of Rio de Janeiro (UFRJ).; 3 Doctorate student in Orthodontics, UFRJ.; 4 Full Professor, Department of Pediatric Dentistry and Orthodontics, UFRJ.

**Keywords:** Biofilms, Orthodontic brackets, *Streptococcus mutans*, Review

## Abstract

**Objective:**

To verify, by means of a systematic review, whether the design of brackets (conventional or self-ligating) influences adhesion and formation of *Streptococcus mutans* colonies.

**Methods:**

Search strategy: four databases (Cochrane Central Register of Controlled Trials, Ovid ALL EMB Reviews, PubMed and BIREME) were selected to search relevant articles covering the period from January 1965 to December 2012. Selection Criteria: in first consensus by reading the title and abstract. The full text was obtained from publications that met the inclusion criteria. Data collection and analysis: Two reviewers independently extracted data using the keywords: conventional, self-ligating, biofilm, *Streptococcus mutans*, and systematic review; and independently evaluated the quality of the studies. In case of divergence, the technique of consensus was adopted.

**Results:**

The search strategy resulted in 1,401 articles. The classification of scientific relevance revealed the high quality of the 6 eligible articles of which outcomes were not unanimous in reporting not only the influence of the design of the brackets (conventional or self-ligating) over adhesion and formation of colonies of *Streptococcus mutans*, but also that other factors such as the quality of the bracket type, the level of individual oral hygiene, bonding and age may have greater influence. Statistical analysis was not feasible because of the heterogeneous methodological design.

**Conclusions:**

Within the limitations of this study, it was concluded that there is no evidence for a possible influence of the design of the brackets (conventional or self-ligating) over colony formation and adhesion of* Streptococcus mutans*.

## Abstract (pt)

**Objetivo:**

verificar, por meio de uma revisão sistemática, se o *design* dos braquetes (convencionais ou autoligáveis) apresenta influência na aderência e formação de colônias de *Streptococcus mutans*.

**Métodos:**

quatro bases de dados (Cochrane Central Register of Controlled Trials; Ovid ALL EMB Reviews; PubMed e BIREME) foram selecionadas para a busca por artigos relevantes, do período de janeiro de 1965 a dezembro de 2012. Os critérios de seleção foram inicialmente aplicados aos títulos e *abstracts* e o texto integral foi obtido de publicações que cumprira os critérios de inclusão. Dois revisores, de forma independente, extraíram os dados utilizando as palavras-chave "convencionais", "autoligados", "biofilme", "*Streptococcus mutans*" e "revisão sistemática" e avaliaram a qualidade metodológica dos estudos incluídos. No caso de divergência, foi adotada a técnica do consenso.

**Resultados:**

a estratégia de busca resultou em 1.401 artigos. A classificação da relevância científica revelou alta qualidade dos 6 artigos elegíveis, cujos desfechos não foram unânimes em relatar a influência do *design* dos braquetes (convencionais ou autoligáveis) sobre a aderência e a formação de colônias de *Streptococcus mutans*, e que outros fatores como características dos tipos de braquetes, o nível de higiene bucal individual, colagem e idade dos indivíduos, podem ter maior influência. O tratamento estatístico foi inviável por causa do desenho metodológico heterogêneo.

**Conclusões:**

dentro das limitações do presente estudo, concluiu-se que não há evidência de uma possível influência do *design* dos braquetes (convencionais ou autoligáveis) sobre a aderência e a formação de colônias de *Streptococcus mutans*.

## INTRODUCTION

Increased oral microbiota of *Streptococcus mutans* and *Lactobacillus* is associated with the onset of tooth demineralization and periodontal disease, especially in orthodontic patients who present greater risk of colonization by these microrganisms.^1-4^ It seems that the main factor behind the increase in the accumulation of dental plaque and inflammatory response is the appearance of new locations of retention around the components of fixed orthodontic appliance.^5^ The devices used in orthodontic appliances (bands, wires, ligatures or brackets) can promote changes in the oral environment, such as pH, amount of *Streptococcus mutans*, biofilm^6-9^ and enamel decalcification.^10-16^ The clinical characteristics and the physical properties of the bracket types are very different,^17^ and, thus, can directly influence the amount of biofilm adhesion and, consequently, gingivitis.^5,18-22^ The characteristics of both the surface of the teeth and the gingiva influence the spontaneous formation of plaque, not only in quantity, but also in quality.^18,23-30^ Saliva composition and secretion rate also influence plaque formation.^27^

Conventional brackets (C) are associated with the use of either elastomeric or stainless steel ligature to keep the orthodontic wire inside the slot.^8^ In Orthodontics, the term self-ligating (SL) refers to orthodontic brackets that have their own mechanism for opening and closing the slot, and do not require any metal or elastomeric ligature as a method for wire ligation.^31,32^ All these methods have advantages and disadvantages, but in relation to biofilm retention, the literature^8,33^ suggests that it is greater with elastomeric ligatures. Orthodontic treatment with C brackets usually presents some periodontal changes as side effects caused by difficulty in periodontal hygiene and also by greater accumulation and qualitative alteration of plaque.^3,5,6,8,19,20^ Thus, in order to improve the deficiency of conventional brackets systems, SL were developed so as to, according to the manufacturers and some studies,^8,34-38^ allow better hygiene. They claim that SL brackets are less susceptible to bacterial colonization due to their shape and absence of elastomeric and metal ligatures.^33^ It is questionable, however, if the adhesion of microorganisms and the development of biofilm is reduced by the removal of ligatures of conventional brackets and with the use of the opening and closing mechanism of SL systems. Even with the changes in modern bracket types, the problem of plaque accumulation around the brackets is still persistent in daily orthodontic practice.^37,39^

Over the years, many publications^6-11,33,34,38-41^ have reported different results concerning microorganism adhesion and biofilm development for C and SL brackets. Biofilm adhesion on brackets is measured by different systems, which hinders the evaluation of scientific quality. Therefore, it was proposed to verify, through a systematic review, whether bracket design (conventional or self-ligating) influences adhesion and formation of *Streptococcus mutans *colonies. Additionally, the methodological soundness of the studies included in the review was assessed in terms of quality.

## MATERIAL AND METHODS

### Search strategy

The strategy of this review was based on the National Health Service Center for Reviews and Dissemination.^42^ Four databases (Cochrane Central Register of Controlled Trials; Ovid ALL EMB Reviews, PubMed and Bireme) were selected to find relevant articles published between January 1965 and December 2012. The search used the keywords "conventional" and/or "self-ligating" crossed with combinations of the terms biofilm and / or *Streptococcus mutans* and / or systematic review. Two reviewers separately sought additional relevant publications, which may not have been in the searched databases, by manually searching for papers in libraries and contacting authors. There were no language restrictions. As a first step, the reviewers selected the articles by reading titles and abstracts. Full texts were obtained from publications that met the inclusion criteria. After the articles were selected, their scientific relevance was independently assessed by the reviewers, and in case of divergence, the technique of consensus was adopted. This review used the PICO (Population Intervention Comparator Outcomes) strategy^43^ to develop both the research and the bibliography (Table 1).

**Table 1:** Description of the PICO (Population Intervention Comparator Outcomes) strategy used to develop the research and the bibliography.

Acronym	Description
Population	Patients with fixed orthodontic appliance with conventional or self-ligating edgewise brackets.
Intervention	Assessment of the amount of biofilm and microbiota attached to conventional or self-ligating brackets.
Comparison	Through the levels of biofilm accumulation on conventional or self-ligating brackets.
Outcomes	Measurement of colonies of Streptococcus mutans and/or their effects on periodontal tissues.

## Inclusion and exclusion criteria

The inclusion criteria for the selected studies initially aimed at human beings, only: those who were periodontally healthy before the study began and who were at 11 years of age or older. The randomized and controlled clinical trials had to involve conventional edgewise and/or self-ligating brackets prescriptions. Case reports, review articles, abstracts and letters to the Editor were also included. The exclusion criteria comprised studies carried out with animals, *in vitro* studies, treatment plans that included extractions of premolars as well as studies that included patients younger than 11 years of age, with periodontal problems, who were users of antibiotics and oral antiseptic solutions, alcoholics and smokers. Articles mentioning patients who used mechanical and anchoring devices, as well as Hyrax, were also excluded.

### Assessment of the scientific relevance of the eligible studies

The following data were collected from each one of the papers selected: author/year of publication, journal, study design, age, teeth involved, bracket type and brand, ligature type, objective and method of analysis, follow-up, statistical analysis and outcome. A quality assessment^44^ was performed on each article, according to the following ten criteria:

1. Study design (randomized clinical trials [RCT], prospective [P] or controlled clinical trials [CCT]) = 2 points.
2. Adequate study description = 1 point.
3. Adequate sample size = 1 point.
4. Adequate sample selection description = 1 point.
5. Drop outs description = 1 point.
6. Adequate description of biofilm measurement method = 0.5 point.
7. Blind study = 0.5 point.
8. Adequate statistics = 1 point.
9. Confounding factors considered = 1 point; and
10. Clinical significance = 1 point.

The ten criteria specified above were used to identify the scientific relevance of the methodological quality of the reviewed papers. The rating was "low" when the points given were less than or equal to 4, "medium" from 5 to 8 points and "high" for 9 or 10 points.

## RESULTS

### Search strategy outcomes

**Figure 1:**
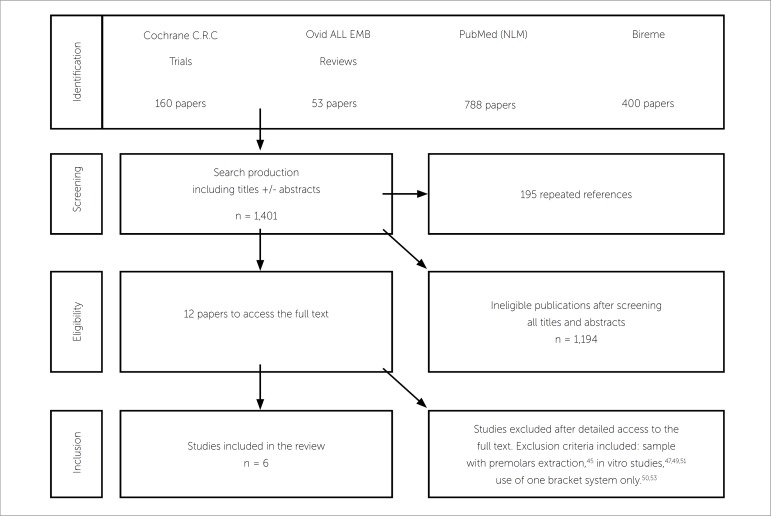
Review flowchart.

The search strategy resulted in 1,401 articles, out of which 195 were repeated references. The exclusion criteria used by both independent reviewers excluded 1,194 articles, which were not considered as relevant to the review, thus, totalizing twelve potentially relevant articles.^33,45-55^ They were chosen for retrieval and evaluation of the full text, for which a summarized data extraction sheet was used (Table 2). Out of the twelve full-text articles that were retrieved, 6 were excluded because: one article^45^ presented premolar extractions in its sample, three^47,49,51^ were *in vitro* studies, and two^50,53^ did not provide a direct comparison between C and SL brackets systems. This resulted in six articles^33,46,48,52,54,55^ that were suitable for the final analysis as they evaluated periodontal and clinical variables originating from bacterial adhesion in patients with C and SL brackets (Fig 1).

**Table 2:** Search data, search strategies and number of results for each database.

Database	Search strategies	Results	Selected papers
Cochrane C.R.C. Trials	conventional OR self-ligating	160	2
Ovid ALL EMB Reviews	exp Orthodontic Appliances / OR edgewise.mp. AND exp Orthodontic Appliance Design/ OR exp Orthodontic Brackets/ OR self-ligating.mp. OR exp Orthodontic Appliances/ AND biofilm.mp. OR exp Dental Biofilm Index/ AND streptococcus mutans.mp. OR exp Streptococcus mutans/	53	4
PubMed (NLM)	conventional AND self-ligating, OR biofilm OR Streptococcus mutans	788	5
Bireme	conventional OR self-ligating	400	1
TOTAL		1,401	12

## Assessment of the scientific relevance of the eligible studies

The six articles^33,46,48,52,54,55^ included in this review (Table 3) met the inclusion criteria, although with differences among their methods of study, sampling, analysis and follow-up. All the eligible studies^33,46,48,52,54,55^ compared both systems: conventional and self-ligating edgewise brackets. Pandis^46^ also made reference to gingival plaque and calculus index, whereas the article by van Gastel^48^ examined the amount of gingival fluid and anaerobic and aerobic colonies. Another study carried out by Pandis^54^ collected saliva 2-3 months after orthodontic appliances had been bonded. *Mitis salivarius* culture medium (MS), specific for *Streptococcus mutans*, was used to count the colony forming units (CFU). Pithon^52^ collected the plaque samples directly from SL and C brackets of different brands, and 3 weeks after bonding, the CFU was carried out in the following culture medium: MS, specific for *S. mutans,* and BHI (Brain Heart Infusion), not specific for bacteria and fungi. In this study,^52^ CFU was visually performed after 24, 48 and 72 hours of incubation. Pejda et al^54^ collected the plaque samples of subgingival sulcus after 18 weeks of treatment, counting 5 periodontal pathogens by PCR, while Pellegrini et al^33^ collected the samples from tooth surfaces surrounding the brackets after 5 weeks of bonding, and the CFU was analyzed by MS and bioluminescence of ATP (adenosine triphosphate).

**Table 3:** Summarized data of the six studies included in the review.

Author Year Journal	Pellegrini et al^33 ^2009 AJODO	Pandis et al^46 ^2008 Orthod Craniofac Res	van Gastel et al^48 ^2007 Journal of Clinical Periodontology	Pithon et al^52 ^2011 Braz J Oral Sci.	Pejda et al^54^ 2012 Angle Orthod	Pandis et al^55 ^2010 Eur J Orthod
Type of study	Randomized controlled trial	Prospective cohort	Randomized controlled trial	Randomized controlled trial	Randomized controlled trial	Randomized controlled trial
Number of patients	18	100	16	5	38	32
Age	11-17 y	12-17 y	17-27 y	20-30 y	11-18 y	11-17 y
Teeth involved	Lateral incisors	Maxilla and mandible	1^st^ and 2^nd^ premolars	Canines; 1^st^ and 2^nd^ premolars and molars (lower)	Maxilla and mandible	Maxilla and mandible
Bracket type/brand	14 p: C – Mini Ovation 14 p: SL – Innovation – R GAC	50 p: C – GAC50 p: SL – In-Ovation – R – GAC	16 C – GAC16 SL – Speed	10 C – Morelli40 SL: GAC; Aditek; Ormco; 3M Unitek	19 p: C – Sprint Forestadent19 p: SL – Damon 3MX, Ormco	16 p: C – GAC16 p: SL – In-Ovation R – GAC
Ligature type	Elastomeric ligatures for the C brackets	Elastomeric ligatures for the C brackets	Elastomeric ligatures for the C brackets	Elastomeric ligatures for the C brackets	Metal ligatures for the C brackets	Elastomeric ligatures for the C brackets
Objective of analysis	Accumulation of bacterial plaque around the brackets. To determine if ATP by bioluminescence may be useful in assessing the plaque index	Index of gingival plaque and calculusof the pocket depth	Crevicular fluid andpocket depth. Aerobic (An) colonies	S. mutans and other microorganisms attachment to C and SL.	Accumulation of different microorganisms on C and SL.	Effect of the type of bracket (C or SL) on the levels of S. mutans in saliva
Method of analysis	MSB specific for S. mutans and determination by bioluminescence	Clinical periodontal parameters	Clinical and microbiological periodontal parameters	MSB specific for S. mutans and BHI, not specific for bacteria and fungus	Clinical periodontal parameters and PCR	MSB specific for S. mutans
Follow-up	5 w	18 m	7 d	21 d	18 w	2-3 m
Statistical analysis	T-tests (1-tailed, with P < 0.05).Chi-squared χ^2^	χ^2^WilcoxonStata	ANOVA Tukey-Kramer	SPSS 13.0Wilcoxon (P < 0.05)	T-testsSidak post hocFisher's tests	ANOVAMinitab 14.20χ^2^
Outcome	SL favor reduced accumulation of S. Mutans and ATP by bioluminescence is useful in assessing plaque index	No advantages of SL over C with respect to the periodontal status of the mandibular anterior teeth	Bracket design can have a significant impact on bacterial load and on periodontal parameters	The hypothesis that self-ligating brackets favor greater aggregation of microorganisms was proved	Bracket design does not seem to have a strong influence on clinical parameters and periodontal pathogens in subgingival plaque.	The total levels of S. mutans do not seem to be significantly different between Cand SL brackets

p = patients y = years m = months w = weeks d = days h = hours C = conventional brackets SL = self-ligating brackets S. = *Streptococcus* SEM = scanning electron microscopy ATP = adenosine triphosphate MSB = Mitis Salivarius agar BHI = brain heart infusion PCR = polymerase chain reaction

When evaluating the scientific relevance of the six eligible articles,^33,46,48,52,54,55^ we found that the description of the sample selection was appropriate, however, the number of drop outs was declared in studies by Pellegrini,^33^ Pandis,^46^ van Gastel^48^ and Pejda^54^. All studies^33,48,52,54^ provided the approval of the Institutional Review Board, except for the articles by Pandis,^46,55^ who asked for the consent of patients / parents before starting the study, only. Considering the confounding factors, similar oral routine and hygiene instructions were given to the subjects taking part in these six studies.^33,46,48,52,54,55^ In the papers,^46,54^ full alignment of the mandibular arch was necessary to eliminate crowding as a confounding factor, but the clinical variables were assessed by the same periodontist. The examiner in the study carried out by Pandis^46^ was not blinded, which could have influenced the outcome of the research, making the results biased. The study conducted by Pithon^52^ did not describe whether it had a blinded examiner, however, as a confounding factor, randomized participants were asked whether they had already received any kind of orthodontic treatment with fixed appliances, since this can have consequences for the smoothness of the tooth enamel and for microbial adhesion at the beginning of biofilm formation.^5,20,21^ All six studies^33,46,48,52,54,55^ used appropriate statistical methods. The examiner's calibration level was reported in one single study,^54^ and only two papers^54,55^ identified the sample calculation. Smoking or medical conditions were clearly identified in studies by van Gastel,^48^ Pejda^54^ and Pandis.^55^ As for the other studies,^33,46,52^ these conditions were declared only after the authors were requested to do so. The final score of the scientific relevance, in accordance with the Jadad scale,^44^ was 10.0 for Pellegrini^33^ and Pejda^54^, 9.5 for van Gastel^48^ and Pandis^55^, and 9.0 for Pandis^46^ and Pithon^52^ (Table 4), which revealed high-quality researches and methodological soundness.

**Table 4:** Quality assessment of the six retrieved studies.

	Pellegrini et al^33^ 2009 AJODO	Pandis et al^46^ 2008 Orthod Craniofac Res	van Gastel et al^48^ 2007 Journal of Clinical Periodontology	Pithon et al^52^ 2011 Braz J Oral Sci.	Pejda et al^54^ 2012 Angle Orthod	Pandis et al^55^ 2010 Eur J Orthod
Type of study	2.0	2.0	2.0	2.0	2.0	2.0
Study description	1.0	1.0	1.0	1.0	1.0	1.0
Sample size	1.0	1.0	1.0	0.5	1.0	1.0
Sample selection description	1.0	1.0	1.0	1.0	1.0	1.0
Drop out description	1.0	0.5	0.5	1.0	1.0	0.5
Measurement method	0.5	0.5	0.5	0.5	0.5	0.5
Blind study	0.5	---	0.5	---	0.5	0.5
Statistics	1.0	1.0	1.0	1.0	1.0	1.0
Confounding factors	1.0	1.0	1.0	1.0	1.0	1.0
Clinical significance	1.0	1.0	1.0	1.0	1.0	1.0
Scale score (Jadad^44^)	10.0	9.0	9.5	9.0	10.0	9.5
Quality standard assessed	high	high	high	high	high	high

### Assessment of the eligible studies outcomes

Among the selected studies, four^46,48,54,55^ had their outcomes consistent in reporting that (a) SL brackets have no advantages over C in periodontal condition of anterior mandibular teeth;^46^ (b) the design of the brackets can have significant impact on bacterial load and periodontal parameters;^48^ and (c) in subgingival plaque and saliva, there seems to be no significant differences in the total levels of *S. Mutans* and periodontal pathogens between C and SL.^54,55^ However, a study^52^ confirmed the hypothesis that SL brackets favor the accumulation of micro-organisms, while another study^33^ reported that SL brackets promote lower retention of *S. mutans* when compared to C (Table 3). The outcomes of the eligible studies^33,46,48,52,54,55^ were not unanimous in reporting that there is evidence of a possible influence of bracket design (conventional or self-ligating) over adhesion and formation of *Streptococcus mutans *colonies.

## DISCUSSION

A systematic review can confirm the quality of a research as well as the methodological soundness of works selected from the literature. Additionally, it can present them for consideration of the clinical and scientific communities. Evidence-based practice requires the construction of a research question and a literature review.

Conventionally, to attach the wire to the brackets, three methods are used: metal ligature, elastomeric ligatures, and the open-close devices of SL brackets. All these methods have advantages and disadvantages, but with regard to the accumulation of biofilm, the literature^8,33^ suggests that elastomeric ligatures favor the retention of biofilm in comparison with the other two methods of ligatures. The question prepared for this review aimed to verify whether bracket design (conventional or self-ligating) influences the formation of *Streptococcus mutans* colonies. Microorganisms exhibit significant adherence to brackets because there are favorable ecological niches in the porous (rough and irregular surfaces of these brackets).^39,47,49,51,56^ Thus, the characteristics of the bracket surface can be considered as harboring favorable sites for the adhesion of biofilm.

### Search strategy outcomes

This research was highly sensitive, addressing evidence of minimum bias. The study carried out by Jordan and LeBlanc^50^ was excluded due to: (a) having assessed one bracket system only, (b) having a not blinded examiner and (c) presenting unspecified statistical analyses. The *in vitro* studies that were excluded^47,49,51^ did not have the inherent characteristics which contribute to the development of intraoral biofilm, and may provide bias results for clinical periodontal conditions.^22^ The differences observed between the results of some papers^33,46,48-50,52^ may be related to factors that include: variations in the shape, material and size between SL and C brackets, the individual level of oral hygiene, salivary flow, treatment variables, types of ligatures, bonding procedures and age of the individuals involved.^24,45,49,51,55^ Thus, bracket type itself would not be a deciding factor for biofilm development, but its composition and material type should be included as factors behind *Streptococcus mutans* colonies formation.^56^

### Assessment of the scientific relevance of the eligible studies

The statistical analysis of our results was not feasible, given that the methodological designs of the eligible articles were heterogeneous. However, the scientific relevance assessment revealed high-quality researches and methodological soundness of all six studies,^33,46,48,52,54,55^ as shown in their final scores, according to the Jadad scale.^44^

Although SL brackets do not require ligatures, their opening and closing mechanism may provide sites for biofilm adhesion similarly to conventional brackets.^46^ This mechanism of SL brackets is not renewed, as it occurs with elastomeric modules in conventional brackets. Moreover, plaque calcification in SL leads to a malfunction of the opening and closing mechanisms. Thus, the theoretical advantages of self-ligating over conventional brackets can be eliminated, as confirmed by other studies.^46,52^ When using conventional brackets, neither the elastomeric rings nor the metal ligatures seem to affect the distribution of bacterial morphotypes in brackets or on the enamel surface.^3^ Aged elastomeric surfaces can apparently favor plaque retention in comparison with polished stainless steel ligatures, but there are no differences between periodontal conditions of patients treated with these two types of ligatures.^8,57^ Nevertheless, some studies^41,58^ report that brackets with elastomeric rings favor damage to gingival conditions, with significant accumulation of biofilm, while the metal ligature had lower retention of biofilm in comparison with other brackets. Some reports^59,60^ affirm that C brackets are directly related to the retention of biofilm, however, the study conducted by Pithon et al^52^ suggests that cross-infection caused by replacement of elastomeric rings is controllable with the use of C brackets, because this type of brackets favors lower formation of *S. Mutans* colonies, which agrees with the study by van Gastel et al^48^ that showed no difference between C and SL in gingival bleeding.

### Assessment of the retrieved studies outcomes

The increase in oral microbiota attachment of *Streptococcus mutans* and *Lactobacillus* is associated with the use of orthodontic appliances,^6,8,9,33,45^ with both C or SL brackets. This increase leads to higher cariogenic plaque, pH low enough to change the clinical periodontal parameters^46,48,54^ and increased risk of enamel demineralization.^6,47^

Some eligible studies^52,54^ evaluated not only the presence of *S. mutans*, but also of other microorganisms related to periodontal disease in patients with C or SL brackets. The study conducted by Pejda et al^54^ found 23.8 times more chance of finding *Aggregatibacter actinomycetemcomitan*s (AA) in subgingival plaque of patients with C brackets, but the increase in AA does not represent a risk factor for local periodontitis, as studies by Paolantonio et al^61,62^ confirm. The differences found between the results of the study by Pithon et al^52^ and the other studies assessed^33,46,48,54,55^ may have been due to methodological differences in some of these studies^46,48,54,55^ in which the CFU were counted from material collected from saliva; Pellegrini et al^33^ collected it from tooth surfaces surrounding the bracket; and, in the study by Pithon,^52^ it was directly collected from the surface of brackets (winglets, slot and cervical region). That was the reason why this latest study should have found statistically significant differences that reveal greater accumulation of biofilm in SL brackets.

### Clinical implications

Some studies^8,33-39^ report that SL brackets are less susceptible to bacterial colonization due to their shape and lack of metal or elastomeric ligatures. However, adequate control of biofilm is more strongly influenced by the correct orientation and cooperation of patients^24,55^ than by simply choosing one system of brackets instead of another. The outcomes of the eligible studies^33,46,48,52,54,55^ were not unanimous in reporting a possible influence of bracket design (conventional or self-ligating) over the adhesion and formation of *Streptococcus mutans *colonies.

The decision of orthodontists on prescribing the use of SL instead of C in their clinical routine, aiming at improving hygiene / plaque accumulation, cannot yet be applied due to lack of scientific evidence.^46,48,52,54,55^ After this review, we presume that there is not enough evidence to support the use of fixed appliances with SL brackets in place of systems with C or vice versa, which agrees with the study by Fleming et al.^63^

Based on the limitations of some works,^64,66^ further studies on other types of brackets, for example, esthetic self-ligating ones, must be performed to visualize the periodontal complications arising from different shapes, sizes and material types of brackets, and with that, guide the development of new systems of brackets design in order to reduce the formation of *Streptococcus mutans *colonies.

## CONCLUSIONS

Within the limitations of this study, it was concluded that there is no evidence for a possible influence of bracket design (conventional or self-ligating) over colony formation and adhesion of *Streptococcus mutans*.

## References

[1] O'Reilly MM, Featherstone JDB (1987). Demineralization and remineralization around orthodontic appliances:
an in vivo study. Am J Orthod Dentofacial Orthop.

[2] Øgaard B, Rølla G, Arends J (1988). Orthodontic appliances and enamel demineralization. Part 1. Lesion
development. Am J Orthod Dentofacial Orthop.

[3] Gwinnett JA, Ceen F (1979). Plaque distribution on bonded brackets. Am J Orthod.

[4] Mizrahi E (1983). Surface distribution of enamel opacities following orthodontic
treatment. Am J Orthod Dentofacial Orthop.

[5] Alexander SA (1991). Effects of orthodontic attachments on the gingival health of permanent
second molars. Am J Orthod Dentofacial Orthop.

[6] Balenseifen JW, Madonia JV (1970). Study of dental plaque in orthodontic patients. J Dent Res.

[7] Menzaghi N, Saletta M, Garattini G, Brambilla E, Strohmenger L (1991). Changes in the yeast oral flora in patients in orthodontic
treatment. Prev Assist Dent.

[8] Forsberg CM, Brattström V, Malmberg E, Nord CE (1991). Ligature wires and elastomeric rings: two methods of ligation, and
their association with microbial colonization of Streptococcus mutans and
lactobacilli. Eur J Orthod.

[9] Rosenbloom RG, Tinanoff N (1991). Salivary Streptococcus mutans levels in patients before, during, and
after orthodontic treatment. Am J Orthod Dentofacial Orthop.

[10] Saemundsson SR, Bergmann H, Magnusdottir MO, Holbrook WP (1992). Dental caries and Streptococcus mutans in a rural child population in
Iceland. Scand J Dent Res.

[11] Sansone C, Van Houte J, Joshipura K, Kent R, Margolis HC (1993). The association of mutans streptococci and non-mutans streptococci
capable of acidogenesis at a low pH with dental caries on enamel and root
surfaces. J Dent Res.

[12] Mattousch TJ, van der Veen MH, Zentner A (2007). Caries lesions after orthodontic treatment followed by quantitative
light-induced fluorescence: a 2-year follow-up. Eur J Orthod.

[13] Mizrahi E (1982). Enamel demineralization following orthodontic
treatment. Am J Orthod.

[14] Benson PE, Parkin N, Millett DT, Dyer FE, Vine S, Shah A (2004). Fluorides for the prevention of white spots on teeth during fixed
brace treatment. Cochrane Database Syst Rev.

[15] Gorelick L, Geiger AM, Gwinnett AJ (1982). Incidence of white spot formation after bonding and
banding. Am J Orthod.

[16] Øgaard B (1989). Prevalence of white spot lesions in 19-year-olds: a study on untreated
and orthodontically treated persons 5 years after treatment. Am J Orthod Dentofacial Orthop.

[17] Anhoury P, Nathanson D, Hughes CV, Socransky S, Feres M, Chou LL (2002). Microbial profile on metallic and ceramic bracket
materials. Angle Orthod.

[18] Loe H, Theilade E, Jensen SB (1965). Experimental Gingivitis in Man. J Periodontol.

[19] Zachrisson S, Zachrisson BU (1972). Gingival condition associated with orthodontic
treatment. Angle Orthod.

[20] Shelley WB (1981). Gingival hyperplasia from dental braces. Cutis.

[21] Theilade E, Wright WH, Jensen SB, Loe H (1966). Experimental gingivitis in man. II. A longitudinal clinical and
bacteriological investigation. J Periodontal Res.

[22] van Pelt AW, Weerkamp AH, Uyen MH, Busscher HJ, de Jong HP, Arends J (1985). Adhesion of Streptococcus sanguis CH3 to polymers with different
surface free energies. Appl Environ Microbiol.

[23] Socransky SS, Haffajee AD, Smith C, Dibart S (1991). Relation of counts of microbial species to clinical status at the
sampled site. J Clin Periodontol.

[24] Quirynen M, Dekeyser C, van Steenberghe D (1991). The influence of gingival inflammation, tooth type, and timing on the
rate of plaque formation. J Periodontol.

[25] Quirynen M, Dekeyser C, van Steenberghe D (1991). Discriminating power of five plaque indices. J Periodontol.

[26] Ramberg P, Axelsson P, Lindhe J (1995). Plaque formation at healthy and inflamed gingival sites in young
individuals. J Clin Periodontol.

[27] Rowshani B, Timmerman MF, Van der Velden U (2004). Plaque development in relation to the periodontal condition and
bacterial load of the saliva. J Clin Periodontol.

[28] Quirynen M, Marechal M, Busscher H, el-Abiad M, Arends J, Van Steenberghe D (1988). The influence of surface characteristics on the early bacterial
colonization of intra-oral hard surfaces. J Clin Dent.

[29] Bollen CM, Quirynen M (1994). Specimen collection in dental plaque and oral
microbiology. Rev Belge Med Dent.

[30] Satou J, Fukunaga A, Satou N, Shintani H, Okuda K (2004). Streptococcal adherence on various restorative
materials. J Oral Rehabil.

[31] Berger J (1999). The engaging concept of self-ligation. Ont Dent.

[32] Cacciafesta V, Sfondrini MF, Ricciardi A, Scribante A, Klersy C, Auricchio F (2003). Evaluation of friction of stainless steel and esthetic self-ligating
bráquetes in various bracket-archwire combinations. Am J Orthod Dentofacial Orthop.

[33] Pellegrini P, Sauerwein R, Finlayson T, McLeod J, Covell DA, Maier T (2009). Plaque retention by self-ligation vs elastomeric orthodontic brackets:
quantitative comparison or oral bacteria detection with adenosine
triphosphate-driven bioluminescence. Am J Orthod Dentofacial Orthop.

[34] Damon DH (1998). The rationale, evolution and clinical application of the self-ligating
bracket. Clin Orthod Res.

[35] Shivapuja PK, Berger J (1994). A comparative study of conventional ligation and self-ligation bracket
systems. Am J Orthod Dentofacial Orthop.

[36] Paduano S, Cioffi I, Iodice G, Rapuano A, Silva R (2008). Time efficiency of self-ligating vs conventional brackets in
orthodontics: effect of appliances and ligating systems. Prog Orthod.

[37] Yu YL, Qian YF (2007). The clinical implication of self-ligating brackets. Shanghai Kou Qiang Yi Xue.

[38] Fernandes C, Almeida R (2008). self-ligating appliances: evolution or revolution?. Aust Orthod.

[39] Türkkahraman H, Sayin MO, Bozkurt FY, Yetkin Z, Kaya S, Onal S (2005). Archwire ligation techniques, microbial colonization, and periodontal
status in orthodontically treated patients. Angle Orthod.

[40] Polson AM, Subtelny JD, Meitner SW, Polson AP, Sommers EW, Iker HP (1988). Long-term periodontal status after orthodontic
treatment. Am J Orthod Dentofacial Orthop.

[41] Garcez AS, Suzuki SS, Ribeiro MS, Mada EY, Freitas AZ, Suzuki H (2011). Biofilm retention by 3 methods of ligation on orthodontic brackets: a
microbiologic and optical coherence tomography analysis. Am J Orthod Dentofacial Orthop.

[42] National Health Service (NHS) Centre for reviews and
dissemination (2001). Undertaking systematic reviews of research on effectiveness [report].

[43] European Food Safety Authority (2010). Application of systematic review methodology to food and feed safety
assessments to support decision making. EFSA J..

[44] Jadad AR, Moore RA, Carrol D, Reynolds DJ, Gavaghan DJ, McQuay HJ (1996). Assessing the quality of reports of randomized clinical trials: is
blinding necessary?. Controlled Clin Trials.

[45] Sukontapatipark W, el-Agroudi MA, Selliseth NJ, Thunold K, Selvig KA (2001). Bacterial colonization associated with fixed orthodontic appliances. A
scanning electron microscopy study. Eur J Orthod.

[46] Pandis N, Vlachopoulos K, Polychronopoulou A, Madianos P, Eliades T (2008). Periodontal condition of the mandibular anterior dentition in patients
with conventional and self-ligating brackets. Orthod Craniofac Res.

[47] Faltermeier A, Burgers R, Rosentritt M (2008). Bacterial adhesion of Streptococcus mutans to esthetic bracket
materials. Am J Orthod Dentofacial Orthop.

[48] van Gastel J, Quirynen M, Teughels W, Coucke W, Carels C (2007). Influence of bracket design on microbial and periodontal parameters in
vivo. J Clin Periodontol.

[49] Brusca MI, Chara O, Sterin-Borda L, Rosa AC (2007). Influence of different orthodontic brackets on Adherence of
microorganisms in vitro. Angle Orthod.

[50] Jordan C, LeBlanc DJ (2002). Influences of orthodontic appliances on oral populations of mutans
streptococci. Oral Microbiol Immunol.

[51] Fournier A, Payant L, Bouclin R (1998). Adherence of Streptococcus mutans to orthodontic
bráquetes. Am J Orthod Dentofacial Orthop.

[52] Pithon MM, Santos RL, Nascimento LE, Ayres AO, Alviano D, Bolognese AM (2011). Do self-ligating brackets favor greater bacterial
aggregation. Braz J Oral Sci.

[53] Ristic M, Vlahovic Svabic M, Sasic M, Zelic O (2007). Clinical and microbiological effects of fixed orthodontic appliances
on periodontal tissues in adolescents. Orthod Craniofac Res.

[54] Pejda S, Varga ML, Milosevic SA, Mestrovic S, Slaj M, Repic d, Bosnjak A (2013). Clinical and microbiological parameters in patients with self-ligating
and conventional brackets during early phase of orthodontic
treatment. Angle Orthod.

[55] Pandis N, Papaioannou W, Kontou E, Nakou M, Makou M, Eliades T (2010). Salivary Streptococcus mutans levels in patients with conventional and
self-ligating brackets. Eur J Orthod.

[56] Carneiro RC (2008). Estudo da microbiota do biofilme supragengival de pacientes em tratamento
ortodôntico com diferentes tipos de braquetes.

[57] Gameiro GH, Nouer DF, Cenci MS, Cury JA (2009). Enamel demineralization with two forms of archwire ligation
investigated using an in situ caries model: a pilot study. Eur J Orthod.

[58] Souza RA, Borges de Araújo Magnani MB, Nouer DF, Oliveira da Silva C, Klein MI, Sallum EA (2008). Periodontal and microbiologic evaluation of 2 methods of archwire
ligation: Ligature wires and elastomeric rings. Am J Orthod Dentofacial Orthop.

[59] Eliades T, Eliades G, Brantley WA (1995). Microbial attachment on orthodontic appliances. I. Wettability and
early pellicle formation on bracket materials. Am J Orthod Dentofacial Orthop.

[60] Batoni G, Pardini M, Ota F, Guica MR, Gabriele M, Campa M, Senesi S (2001). Effect of removable orthodontic appliances on oral colonization by
mutans streptococci in children. Eur J Oral Sci.

[61] Paolantonio M, Festa F, di Placido G, D'Attilio M, Catamo G, Piccolomini R (1999). Site-specific subgingival colonization by Actinobacillus
actinomycetemcomitans in orthodontic patients. Am J Orthod Dentofacial Orthop.

[62] Paolantonio M, Pedrazzoli V, di Murro C, di Placido G, Picciani C, Catamo G (1997). Clinical significance of Actinobacillus actinomycetemcomitans in young
individuals during orthodontic treatment. A 3-year longitudinal
study. J Clin Periodontol.

[63] Fleming PS, Johal A (2010). Self-ligating brackets in orthodontics. Angle Orthod.

[64] Maza JL, Elguezabal N, Prado C, Ellacuria J, Soler I, Ponton J (2002). Candida albicans adherence to resin-composite restorative dental
material: influence of whole human saliva. Oral Surg Oral Med Oral Pathol Oral Radiol Endod.

[65] Addy M, Shaw WC, Hansford P, Hopkins M (1982). The effect of orthodontic appliances on the distribution of Candida
and plaque in adolescents. Br J Orthod.

[66] Park JH, Gakunga PT, Aemechi BT (2007). Influence of self-ligating orthodontic brackets on plaque accumulation
in vitro. J Dent Res.

